# Meta-review of the effectiveness of computerised CBT in treating depression

**DOI:** 10.1186/1471-244X-11-131

**Published:** 2011-08-12

**Authors:** Pooria Sarrami Foroushani, Justine Schneider, Neda Assareh

**Affiliations:** 1 CLAHRC-NDL, The University of Nottingham, School of Community Health Sciences, Sir Colin Campbell Building, Jubilee campus, Triumph Road, Nottingham, NG8 1BB, UK; 2 CLAHRC-NDL, The University of Nottingham, School of Sociology and Social Policy, University Park, Nottingham, NG7 2RD, UK; 3 The University of Nottingham, School of Biomedical Sciences, NG7 2UH, UK

## Abstract

**Background:**

Several computerised cognitive behaviour therapy (cCBT) packages are now available to treat mild to moderate depression with or without anxiety. These have been usually been reviewed alongside cCBT for a wide range of psychological problems. Here, we single out the results of these reviews for the most common mental disorder, mild to moderate depression. The aim of this paper is to evaluate the quality of existing reviews and to enable reliable comparisons of alternative computer packages for the same patient group.

**Methods:**

A thorough search and analysis of reviews of efficacy of cCBT published between 1999 and February 2011.

**Results:**

The search yielded twelve systematic reviews from ten studies covering depression. Their methodology is appraised and selected findings are presented here.

**Conclusions:**

The meta-review supports the efficacy of cCBT for treatment of depression; however there is limited information on different approaches, whose relative cost-effectiveness remains to be demonstrated. Suggestions are made for future studies in the field.

## Background

Depression and anxiety are amongst the most prevalent psychiatric conditions, affecting up to 8.8% of the population in the UK [1]. It is estimated that depression in adults could cost the nation over £9 billion a year and could lead to 109.7 million working days lost per year [2]. To address the problem, there is a strong evidence base for psychological therapy featuring cognitive behaviour therapy (CBT) [3]. However, conventional CBT is delivered face-to-face, meaning that the resources which would be needed to treat everyone who could potentially benefit for depression are vast.

The past decade has seen growing use of computers and the internet as an adjunct to face-to-face CBT, and in some cases as an alternative to individual contact with a clinician. A computer can be used in different ways; to provide information in written or audio/video formats; as a tool for screening or diagnosis; as a self-help tool, which entails following a structured programme on-line; to simulate situations and permit graded exposure in highly controlled conditions; and as a means of communication between patients and health care professionals for administrative or therapeutic purposes. The attractions of computer-mediated CBT (cCBT) include: its capacity to deliver structured input consistently with precision, low cost, easy accessibility and potential to offer a flexible approach in a non-stigmatising environment. Yet if best use of this resource is to be made, clear knowledge about the effectiveness and costs of alternative cCBT programmes is needed to guide planning and provision of care.

The main focus of this paper is on the delivery of computerised CBT (cCBT) for treating depression with or without anxiety. From a pragmatic perspective, users, clinicians and commissioners of health care are most likely to ask 'what works for people with depression?'. Since we were engaged in a study of MoodGYM, which is a publicly-available cCBT programme developed by the Centre for Mental Health Research at the Australian National University [4,5], we wanted to place this study in the context of relevant literature concerning both MoodGYM and other similar programmes which are also primarily designed to treat depression. There have been different reviews on the topic, but their quality and comprehensiveness of their scope has not been studied yet. We therefore set out to review existing reviews, appraise these and draw together what they indicate regarding cCBT for depression, particularly if their results could help us to compare alternative cCBT packages for the same disorder.

## Methods

The search strategy is given in the Additional file 1. The authors collaborated in the review and searched the following electronic data-bases: Medline, Embase, CINAHL (EBSCO), the British Nursing Index, the Cochrane Library, and PsycINFO. All systematic reviews examining efficacy of cCBT for depression with or without anxiety in adults were included at the outset, limited to the last ten years (initially searching 1999 to 2009, then updating to Feb 2011). The evolutionary development of cCBT and its reliance on ever-increasing speeds of internet communications make earlier reviews unlikely to be relevant to our aim. We also considered papers that were suggested to us via personal communications. We excluded books, chapters, commentaries and reviews whose methodology was not systematic or reviews whose methodology could not be ascertained, as well as systematic reviews that did not directly address effectiveness, e.g. those looking at patient acceptability or drop-out alone. To verify the methodology of articles, authors were consulted via email when necessary. The search was restricted to publications in English, since the cCBT packages of interest use the English language. The PRISMA statement guided the approach [6] and the Critical Appraisal Skills Programme (CASP) tool [7] was used to extract data from the papers identified.

## Results

**Figure 1:**
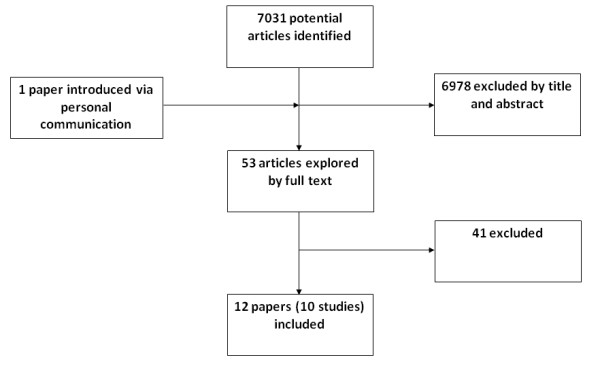
**Summary of study selection and exclusion**.

The initial search identified 7031 studies, from which 6978 were excluded by screening the title and abstract. The remaining 52 papers were obtained and read; as a result 41 were excluded. Additional file 2 lists these and the reasons why. One paper identified through personal communication was included in the review. Twelve papers covering ten reviews of the effectiveness of cCBT for depression with or without anxiety in adults were included (Table 1 and additional file 3), and are summarised below (Figure 1).

**Table 1:** summarising results of the included papers

Authors/year	Results and conclusions
**Kaltenthaler et al., 2002 **[8]**; 2004 **[9]	**MoodGYM: **Package not directly discussed in the review.
	**ODIN: **Package not directly discussed in the review.
	**Colour Your life: **Package not directly discussed in the review.
	**Overcoming Depression: **One study reported, which concluding cCBT was not better than TAU.
	**Beating the Blues: **Three RCTs reported, in one of them some immediate improvement were seen one month after treatment, but not after 3 and 6 months, while the other studies indicated continuation of benefits.
	**General conclusion of the review: **CCBT is as effective as TCBT (poor-to-moderate quality evidence); CCBT is more effective than TAU (limited evidence of poor-to-moderate quality); CCBT may be as effective as or less effective than bibliotherapy; CCBT might reduce therapist time; CCBT could be useful in a stepped-care programme; No study is available on economic analysis of CCBT.

**Kaltenthaler et al., 2006 **[9]	**MoodGYM: **based on one study, MoodGYM reported to be effective in reducing symptoms of depression.
**Kaltenthaler, et al, 2008 **[11]	**ODIN: **In one study it was reported to have no significant impact, rather than a moderated effect on people with low level of depression, in another study, reduction of depression scores were observed.
	**Colour Your life: **Package not directly discussed in the review.
	**Overcoming Depression: **No RCT evidence for Overcoming Depression is available, but based on one published study, there was improvement in depression symptoms.
	**Beating the Blues: **Based on RCT studies as well as non-comparative studies, Beating the Blue is effective.
	**General conclusion of the review: **There is some evidence that CCBT is more effective than TAU in the treatment of depression/anxiety; CCBT might reduce therapist time compared with TCBT; There is some evidence to support the effectiveness of CCBT for the treatment of depression; All studies were associated with considerable drop-out rates; Little evidence was presented regarding participants' preferences and the acceptability of the therapy.

**Griffiths and Christensen, 2006 **[14]	**MoodGYM: **based on two studies, MoodGYM reported to be effective in reducing symptoms of depression.
	**ODIN: **one study it was reported to have no significant impact.
	**Colour Your life: **Package not directly discussed in the review.
	**Overcoming Depression: **Package not directly discussed in the review.
	**Beating the Blues: **Package not directly discussed in the review.
	**General conclusion of the review: **The overall conclusion is based on different interventions and health problems. It was concluded that most interventions were reported to be effective in reducing risk factors or improving symptoms, and it was suggested that many of the studies had methodological limitations.

**Spek et al, 2007 **[12]	**MoodGYM: **Package not directly discussed in the review.
	**ODIN: **Package not directly discussed in the review.
	**Colour Your life: **Package not directly discussed in the review.
	**Overcoming Depression: **Package not directly discussed in the review.
	**Beating the Blues: **Package not directly discussed in the review.
	**General conclusion of the review: **the review is a meta-analysis on treatment of depression and anxiety that indicates moderate to large mean effect size; significant heterogeneity was reported.

**Andersson 2009 **[13]	**MoodGYM: **Package not directly discussed in the review.
	**ODIN: **Package not directly discussed in the review.
	**Colour Your life: **Package not directly discussed in the review.
	**Overcoming Depression: **Package not directly discussed in the review.
	**Beating the Blues: **Package not directly discussed in the review.
	**General conclusion of the review: **Although there are limitations in evidences, internet-based and computer based treatments seems to be effective in treatment of people with depression.

**Griffiths et al, 2010 **[15]	**MoodGYM: **Package not directly discussed in the review.
	**ODIN: **Package not directly discussed in the review.
	**Colour Your life: **Package not directly discussed in the review.
	**Overcoming Depression: **Package not directly discussed in the review.
	**Beating the Blues: **Package not directly discussed in the review.
	**General conclusion of the review: **6/8 trials yielded CBT has positive effects on depression. Authors concluded that internet interventions offer promise for use.

**García-Lizana and Muñoz-**	**MoodGYM: **Package not directly discussed in the review.
**Mayorga 2010 **[16]	**ODIN: **Package not directly discussed in the review.
	**Colour Your life: **Package not directly discussed in the review.
	**Overcoming Depression: **Package not directly discussed in the review.
	**Beating the Blues: **Package not directly discussed in the review.
	**General conclusion of the review: **Authors concluded that there is insufficient evidence for the effectiveness of ICT use in treatment of depression. However, they based their conclusions on various and heterogeneous interventions.

**Andrews et al 2010 **[17]	**MoodGYM: **Package not directly discussed in the review.
	**ODIN: **Package not directly discussed in the review.
	**Colour Your life: **Package not directly discussed in the review.
	**Overcoming Depression: **Package not directly discussed in the review.
	**Beating the Blues: **Package not directly discussed in the review.
	**General conclusion of the review: **cCBT was suggested to be effective and acceptable for treatment of depression and anxiety.

**Wade 2010 **[18]	**MoodGYM: **based on three studies, MoodGYM reported to be effective in reducing symptoms of depression.
	**ODIN: **In one study it was reported to have no significant impact, in another study, statistically significant small effect size in reduction of depression scores was observed.
	**Colour Your life: **based on one study, this programme could improve depressive symptoms.
	**Overcoming Depression: **Package not directly discussed in the review.
	**Beating the Blues: **Package not directly discussed in the review.
	**General conclusion of the review: **the review is concerned with different applications of the internet, and it was suggested that the internet has potentials for supporting patients with depression.

**Titov 2011 **[19]	**MoodGYM: **based on one study on MoodGYM, having multiple CBT components is associated with a better outcome.
	**ODIN: **Package not directly discussed in the review.
	**Colour Your life: **Package not directly discussed in the review.
	**Overcoming Depression: **Package not directly discussed in the review.
	**Beating the Blues: **Package not directly discussed in the review.
	**General conclusion of the review: **There is evidence for progression in the field of internet-delivered psychotherapy.

(cCBT: Computerised Cognitive Behaviour Therapy; ICT: Information and communication technology; RCT: Randomised Control Trials; TAU: Treatment as usual; TCBT: Therapist-led cognitive behaviour therapy)

### Included reviews

Kaltenthaler et al. [8,9] undertook two reviews for the National Institute of Health Research Health Technology Assessment Board; these comprised a wide range of computerised interventions based on CBT, and were also the basis for two other publications [10,11]. They met all the criteria for inclusion in this meta-review, more than any of the other papers examined.

Spek et al [12] performed a meta-analysis to explore the effectiveness of internet-based CBTs. Although they searched only three electronic data bases, they identified and carefully investigated 12 RCTs. They admit that their results could be affected by the heterogeneity of studies. Similarly Andersson and Cuijpers [13] performed a meta-analysis on 12 RCTs to evaluate internet-based and computer-based treatments for depression in adults with depression, and they have also concerns about heterogeneity of treatments and samples.

Griffiths and Christensen [14] looked at all Internet interventions for mental disorders. They identified 15 RCTs, from which only three were related to depression, and one of these was undertaken by Christensen and Griffiths. The latter reported positive results for cCBT, but the other two studies did not. Nevertheless, Griffiths and Christensen concluded overall that RCTs have shown improvement in symptoms of depression. Griffiths and Christensen are also authors of the MoodGYM-related articles which they reviewed [14,15].

Four other reviews [15-18] had a wider scope than cCBT for depression. They include other uses of the internet (e.g. for screening and educating), other interventions (e.g. internet based therapies), other health problems (e.g. anxiety and panic disorder), and other evaluative aspects (e.g. acceptability). One of these reviews had a search strategy that was limited to Medline [18]. The most recent published review was undertaken by Titov [19], who summarized 13 RCTs and explored developments in the field of internet-delivered psychotherapies for depression.

Although it is desirable to compare different cCBT programmes and to summarise the reviews' findings in relation to different packages, there are considerable impediments to this (see table 1). Four reviews did not name the packages they examined [12,15-17]. Where a review specifies packages for a specific disorder, it may not include all the available packages due to its limited search strategy [18], or more recent packages such as 'Colour Your Life', [8-11]. As highlighted by Kaltenthaler et al [9], most studies on depression-related packages were tested by non-comparative trials (i.e. according to improvement in individual measures against their baseline) rather than by comparison with alternative approaches.

### Methodology of reviews

As discussed above, the reviews varied in their rigour and scope (Additional file 3). The number of electronic data-bases included varied from 1 to 17 (median: 5). The most commonly-searched databases were Medline, PsycINFO and the Cochrane Library. Reviews also varied in terms of the intervention programmes and target populations covered. Some had a broader definition of the intervention than cCBT; for example they looked at 'self-help approaches' [14]. The diagnosis of interest of one review was depression only [16], but others included conditions such as anxiety disorders or broader categories, such as 'mental health problems'; thus results include for example obsessive compulsive disorder, insomnia and eating disorders [14]. There was an average of two years between the searches and the publication dates of the reviews, which is a particular limitation in a rapidly-advancing field [20].

### Economic aspects

It is important to bear in mind that the price of an intervention is only one variable amongst several that determine its cost-effectiveness. The cost-effectiveness of cCBT depends on its results relative to the likely alternative treatment (or none), as well as on the costs of each option. Thus, any cCBT package which reduces therapist time, while being no less effective than therapist-led treatment, is likely to be cost-effective unless it costs more to use the cCBT than the time saved. To illustrate, the cost of one package, Beating the Blues, per quality-adjusted life-year is estimated to be £1250. This estimate comes from what, according to Kaltenthaler et al [9], is the only published paper on cost effectiveness of cCBT. However, the same review excluded 'commercially-sensitive' information, which highlights the fact that many cCBT packages compete in a market for sales and profits. For this reason, we may never have access to the information needed to compare the relative cost-effectiveness of rival packages.

### Clinical effectiveness

As previously mentioned, reviews do not universally refer to the individual packages and reported studies are not always comparative. Together these factors make comparison of packages difficult. However, based on the available information, reviews have concluded that MoodGYM was effective in reducing symptoms of depression [9,11,13,14,18,19], ODIN had moderate or no significant effect [9,11,14,18], there is not enough data with good quality to support Overcoming Depression [8-11], Beating the Blues is judged to be effective [8-11], and 'Colour Your Life' is also reported to improve symptoms of depression [18].

Although there is limited information in the reviews that would permit comparison of individual packages, their results broadly agree that, for depression and anxiety disorders, cCBT has been found to be no less effective than therapist-led cognitive behaviour therapy (TCBT) [8-10]. Moreover, cCBT has been shown to reduce therapist time [8,9], be more effective than 'treatment as usual' [8-10], and equally or less effective than bibliotherapy [8,10]. There is also good evidence that self-help internet interventions improve symptoms [14], although the effect size was larger for anxiety than for depression [12].

## Discussion

Having critically appraised the quality of several reviews, we had to exclude 22 reviews that did not describe their methodology. Thirty-one other papers were excluded for reasons such as having a focus other than efficacy of cCBT on depression. Even the included reviews provided minimal information about individual packages, and their conclusions were based on only a small number of RCTs on each individual package (table 1). In effect we had to unpick even the better reviews to arrive at a conclusion about cCBT for depression.

By singling out the evidence pertaining to depression we have revealed reasonably consistent inferences drawn by from ten systematic reviews (twelve papers) of varying quality and relevance. Bearing in mind the (declared) interests of certain authors and the largely uncritical approach taken by some reviewers, we cautiously infer that certain cCBT packages, specifically MoodGYM, Beating the Blues and Colour Your Life, can have a positive effect on symptoms of depression. Yet there is limited information available to compare alternative packages, which is an important challenge given the differences in costs. The finding that cCBT reduces therapist time could have important implications for service organisation and costs, but the effect size of each intervention as well as the costs need to be compared before superior efficacy for cCBT can be claimed, which highlights the need for high-quality comparative studies as new packages are developed.

## Conclusions

At present there is limited evidence which indicates the effectiveness of MoodGYM, Beating the Blues and Colour Your Life, but not enough of such evidence to prefer one package for depression over another. Two studies currently in the field will have an important bearing on this position. The Randomised Evaluation of the Effectiveness and Acceptability of Computerised Therapy (REEACT) Trial is due to end in 2013 (ISRCTN91947481) and Computerised CBT for Common Mental Disorders: RCT of a Workplace Intervention (ISRCTN 24529487) concludes in August, 2011. They will partly meet the need highlighted here for more RCTs on specific cCBT packages, both in relation to outcomes and in relation to costs. As new packages become available, their effectiveness will also need to be established, particularly if they charge for use.

## List of abbreviations

CASP: (Critical Appraisal Skills Programme); CBT: (Cognitive Behaviour Therapy); cCBT: (Computerised Cognitive Behaviour Therapy); ICT: (Information and Communication Technology); RCT: (Randomised Control Trials); TAU: (Treatment As Usual); TCBT: (Therapist-led Cognitive Behaviour Therapy).

## Competing interests

Justine Schneider is the chief investigator, and Pooria Sarrami was the trial coordinator of a study on effectiveness of cCBT for treatment of depression, anxiety and stress in workplaces, funded by the British Occupational Health Research Foundation.

## Authors' contributions

PS contributed in searching and screening of papers, data extraction, producing summary tables and writing up; JS contributed in data extraction and writing up and NA contributed to the searching and screening of papers. All authors read and approved the final manuscript.

## Pre-publication history

The pre-publication history for this paper can be accessed here:

http://www.biomedcentral.com/1471-244X/11/131/prepub

## Supplementary Material

Additional file 1 **Search strategy**. This file describes the key terms and the combinations used for the search. Click here for file

Additional file 2 **Excluded reviews**. This file lists excluded reviews and explains the reason for exclusion. Click here for file

Additional file 3 **Included reviews**. This file lists included review and provides details of their methodology and scope. Click here for file
