# Supplementary material for: Meta-review of the effectiveness of computerised CBT in treating depression
Source: BMC Psychiatry. 2011 Aug 12;11:131. doi: 10.1186/1471-244X-11-131 (PMC3180363; doi:10.1186/1471-244X-11-131)
Supplement: Additional file 1 — Search strategy. This file describes the key terms and the combinations used for the search. [file 1471-244X-11-131-S1.DOC]

**Search strategy**

1 = computer*

2 = online*

3 = internet*

4 = remote

5 = e-health

6 = website

7 = virtual therapist

8 = 1 or 2 or 3 or 4 or 5 or 6 or 7

9 = cognitive*

10 = CBT

11 = psychotherapy

12 = psychological treatment

13 = 9 or 10 or 11 or 12

14 = depression

15= MoodGYM

16 = overcoming depression

17 = beating the blues

18 = 15 or 16 or 17

**Final**: (8 and 13 and 14) or 18
