# Supplementary material for: Meta-review of the effectiveness of computerised CBT in treating depression
Source: BMC Psychiatry. 2011 Aug 12;11:131. doi: 10.1186/1471-244X-11-131 (PMC3180363; doi:10.1186/1471-244X-11-131)
Supplement: Additional file 2 — Excluded reviews. This file lists excluded reviews and explains the reason for exclusion. [file 1471-244X-11-131-S2.DOC]

# Excluded reviews

| **Authors / year** | **The intervention** | **Target condition** | **Reason for exclusion** |
| --- | --- | --- | --- |
| Finfgeld, 1999 [1] | Computer assisted therapy | Mental health problems | Limited information available on the method of the review. |
| Marks, 2000 [2] | Psychosocial treatments (computer self-help systems, etc) | Anxiety disorders, depression, etc. | Limited information available on the method of the review. |
| Proudfoot, 2004 [3] | Computer based treatments | Anxiety and depression | Not a systematic review, as also confirmed by the author. |
| Cavanagh and Shapiro, 2004 [4] | Computer treatments | Common mental health problems (depression and anxiety) | Limited information available on the method of the review. |
| Anderson et al 2004 [5] | Computer supported cognitive-behavioural treatment including palmtop computers, virtual reality exposure therapy, and personal computer software programmes. | anxiety disorders | Limited information available on the method of the review. |
| Emmelkamp, 2005 [6] | application of computer technology and the use of the Internet | mental health care (anxiety disorders, mood disorders, and substance abuse disorders) | Limited information available on the method of the review. |
| Pull 2006 [7] | Self-help internet interventions | Mental disorders (depressive disorders, anxiety disorders, substance use disorders, insomnia, tinnitus) | Limited information available on the method of the review. |
| Hirai and Clum 2006 [8] | Self help approaches | Anxiety problems | Depression not included. |
| Titov, 2007 [9] | Computerized cognitive behavioural therapy | Anxiety and depression | Limited information available on the method of the review. |
| Peck, 2007 [10] | Computer-guided cognitive behavioural therapy | Anxiety disorders | Limited information available on the method of the review. |
| Griffiths and Christensen, 2007[11] | Internet-based cognitive behaviour therapy (CBT) programmes | Depression and anxiety | Limited information available on the method of the review. |
| Lauder et al, 2007 [12] | Psychosocial interventions | Psychiatric disorders | Limited information available on the method of the review. |
| Palmqvist, et al 2007 [13] | Internet delivered cognitive behavioural treatment | Mood and anxiety disorders | Not a systematic review, as also confirmed by the author. |
| Christensen, 2007 [14] | Computerised therapy | psychiatric disorders | A commentary article. |
| Postel 2008 [15] | E-therapy(internet-based intervention) | Mental health problems (reviewing only two studies on depression) | Focus is on the quality of RCTs; not the effectiveness |
| Andersson and Cuijpers, 2008 [16] | Online cognitive–behavioural therapy (CBT) | Depression | Not a systematic review, as also confirmed by the author. |
| Morgan and Jorm, 2008 [17] | Complementary and self-help interventions | Depressive disorders | The section on computerised interventions was based on a previous review (Spek et al, 2007) [18], which is already included in this meta-review. |
| Ahmead and Bower 2008 [19] | Self help materials, | Depression, anxiety, self concept, self esteem, self efficacy, locus of control | Age group not adults. |
| Kaltenthaler et all 2008 [20] | Computerized cognitive behaviour therapy | Depression | Focus is on acceptability; not the effectiveness. |
| Bryant, 2008 [21] | E-learning | Primary care patients | Limited information available on the method of the review, as also confirmed by the author. |
| Marks and Cavanagh, 2009 [22] | Computer-aided psychotherapy | Mental health problems | Limited information available on the method of the review. |
| Andersson and Carlbring, 2009 [23] | Computer-based psychological treatments | Patients | A commentary article. |
| Andersson and Carlbring, 2009 [24] | Internet treatment | social anxiety disorder (social phobia) | A commentary article. |
| Marks et al, 2009 [25] | Meta-analysis of computer-aided psychotherapy | Anxiety disorders | Limited information available on the method of the review. |
| Reger and Gahm, 2009 [26] | Internet- or computer-based treatment (ICT) | Anxiety | Depression not included. |
| Waller and Gilbody 2009 [27] | CCBT | Anxiety or depression | Focus is on the uptake, not effectiveness. |
| Cuijpers et al, 2009 [28] | Computer-aided psychotherapy (CP) | phobias; panic disorder/agoraphobia; PTSD; obsessive–compulsive disorder | Depression not included. |
| Calear and Cristensen, 2010[29] | Internet-based programs | Anxiety and depression | Age group not adults. |
| Abbas and Driessen, 2010 [30] | Short-Term Psychodynamic  Psychotherapy (STPP) for depression | Depression | A commentary article. |
| Miclea et al, 2010  [31] | Computer-mediated psychotherapy | Mental health | Limited information available on the method of the review. |
| Kaltenthaler and Cavanagh, 2010  [32] | cCBT | Mental health disorders | Limited information available on the method of the review. |
| Cuijpers et al, 2010  [33] | CBT | depression | Computerised/internet-based cbt is not explored. |
| Peck, 2010  [34] | Computerised self help | Mental health | Not a systematic review, as also confirmed by the author. |
| Titov et al, 2010  [35] | cCBT | Mental health | Limited information available on the method of the review. |
| Spurgeon and Wright, 2010  [36] | cCBT | Psychiatric conditions | Limited information available on the method of the review. |
| Richardson et al, 2010  [37] | cCBT | Depression and anxiety | Age group not adults. |
| Andrews, 2010  [38] | cCBT | Depression | Limited information available on the method of the review (editorial). |
| Arroll and Moir, 2010  [39] | Treatment in primary care | depression | Limited information available on the method of the review (editorial). |
| Carroll and Rounsaville, 2010  [40] | Computer-assisted therapy | Mental health | Limited information available on the method of the review. |
| Cartreine et al, 2010  [41] | Computer-based psychotherapy | Psychiatry and behavioural health | Limited information available on the method of the review. |
| Newman et al, 2011  [42] | Technology-assisted self help | Anxiety and depression | Limited information available on the method of the review. |

## 1. Finfgeld, D.L., *Computer-assisted therapy: harbinger of the 21st century?* Archives of Psychiatric Nursing, 1999. 13(6): p. 303-10.

## 2. Marks, I., *Forty years of psychosocial treatments.* Behavioural and Cognitive Psychotherapy, 2000. 28(4): p. 323-334.

## 3. Proudfoot, J.G., *Computer-based treatment for anxiety and depression: Is it feasible? Is it effective?* Neuroscience and Biobehavioral Reviews, 2004. 28(3): p. 353-363.

## 4. Cavanagh, K. and D.A. Shapiro, *Computer treatment for common mental health problems.* Journal of Clinical Psychology, 2004. 60(3): p. 239-51.

## 5. Anderson, P., C. Jacobs, and B.O. Rothbaum, *Computer-Supported Cognitive Behavioral Treatment of Anxiety Disorders.* Journal of Clinical Psychology, 2004. 60(3): p. 253-267.

## 6. Emmelkamp, P.M.G., *Technological innovations in clinical assessment and psychotherapy.* Psychotherapy and Psychosomatics, 2005. 74(6): p. 336-343.

## 7. Pull, C.B., *Self-help Internet interventions for mental disorders.* Current Opinion in Psychiatry, 2006. 19(1): p. 50-53.

## 8. Hirai, M. and G.A. Clum, *A Meta-Analytic Study of Self-Help Interventions for Anxiety Problems.* Behavior Therapy, 2006. 37(2): p. 99-111.

## 9. Titov, N., *Status of computerized cognitive behavioural therapy for adults.* Australian & New Zealand Journal of Psychiatry, 2007. 41(2): p. 95-114.

## 10. Peck, D., *Computer-guided cognitive-behavioural therapy for anxiety states.* Psychiatry, 2007. 6(4): p. 166-169.

## 11. Griffiths, K.M. and H. Christensen, *Internet-based mental health programs: a powerful tool in the rural medical kit.* Australian Journal of Rural Health, 2007. 15(2): p. 81-87.

## 12. Lauder, S., A. Chester, and M. Berk, *Net-effect? Online psychological interventions.* Acta Neuropsychiatrica, 2007. 19(6): p. 386-388.

## 13. Palmqvist, B., P. Carlbring, and G. Andersson, *Internet-delivered treatments with or without therapist input: Does the therapist factor have implications for efficacy and cost?* Expert Review of Pharmacoeconomics and Outcomes Research, 2007. 7(3): p. 291-297.

## 14. Christensen, H., *Computerised therapy for psychiatric disorders.* Lancet, 2007. 370(9582): p. 112-113.

## 15. Postel, M.G., H.A. De Haan, and C.A.J. De Jong, *E-therapy for mental health problems: A systematic review.* Telemedicine and e-Health, 2008. 14(7): p. 707-714.

## 16. Andersson, G. and P. Cuijpers, *Pros and cons of online cognitive-behavioural therapy.* British Journal of Psychiatry, 2008. 193(4): p. 270-271.

## 17. Morgan, A.J. and A.F. Jorm, *Self-help interventions for depressive disorders and depressive symptoms: A systematic review.* Annals of General Psychiatry, 2008. 7(14).

## 18. Spek, V., et al., *Internet-based cognitive behaviour therapy for symptoms of depression and anxiety: A meta-analysis.* Psychological Medicine, 2007. 37(3): p. 319-328.

## 19. Ahmead, M. and P. Bower, *The effectiveness of self help technologies for emotional problems in adolescents: A systematic review.* Child and Adolescent Psychiatry and Mental Health, 2008. 2(20).

## 20. Kaltenthaler, E., et al., *The acceptability to patients of computerized cognitive behaviour therapy for depression: A systematic review.* Psychological Medicine, 2008. 38(11): p. 1521-1530.

## 21. Bryant, S.L., *E-learning for patients.* Education for Primary Care, 2008. 19(2): p. 124-129.

## 22. Marks, I. and K. Cavanagh, *Computer-aided psychological treatments: evolving issues.* Annual Review of Clinical Psychology, 2009. 5: p. 121-41.

## 23. Andersson, G. and P. Carlbring, *Moving on to comorbidity, new modes of delivery and acceptability.* Addiction, 2009. 104(3): p. 389-390.

## 24. Andersson, G. and P. Carlbring, *Commentary on Berger, Hohl, and Caspar's (2009) Internet-based treatment for social phobia: a randomized controlled trial.* Journal of Clinical Psychology, 2009. 65(10): p. 1036-8.

## 25. Marks, I.M., et al., *Meta-analysis of computer-aided psychotherapy: Problems and partial solutions.* Cognitive Behaviour Therapy, 2009. 38(2): p. 83-90.

## 26. Reger, M.A. and G.A. Gahm, *A meta-analysis of the effects of Internet-and computer-based cognitive-behavioral treatments for anxiety.* Journal of Clinical Psychology, 2009. 65(1): p. 53-75.

## 27. Waller, R. and S. Gilbody, *Barriers to the uptake of computerized cognitive behavioural therapy: A systematic review of the quantitative and qualitative evidence.* Psychological Medicine, 2009. 39(5): p. 705-712.

## 28. Cuijpers, P., et al., *Computer-aided psychotherapy for anxiety disorders: A meta-analytic review.* Cognitive Behaviour Therapy, 2009. 38(2): p. 66-82.

## 29. Calear, A.L. and H. Christensen, *Review of internet-based prevention and treatment programs for anxiety and depression in children and adolescents.* Medical Journal of Australia, 2010. 192(11 Suppl): p. S12-4.

## 30. Abbass, A. and E. Driessen, *The efficacy of short-term psychodynamic psychotherapy for depression: A summary of recent findings.* Acta Psychiatrica Scandinavica, 2010. 121 (5): p. 398.

## 31. Miclea, M., et al., *Computer-mediated psychotherapy. Present and prospects. A developer perspective.* Cognition, Brain, Behavior: An Interdisciplinary Journal, 2010. 14: p. 185-208.

## 32. Kaltenthaler, E. and K. Cavanagh, *Computerised cognitive behavioural therapy and its uses.* Progress in Neurology and Psychiatry, 2010. 14: p. 22-29.

## 33. Cuijpers, P., et al., *Efficacy of cognitive-behavioural therapy and other psychological treatments for adult depression: meta-analytic study of publication bias.* British Journal of Psychiatry, 2010. 196: p. 173-8.

## 34. Peck, D.F., *The therapist-client relationship, computerized self-help and active therapy ingredients.* Clinical Psychology & Psychotherapy, 2010. 17: p. 147-153.

## 35. Titov, N., G. Andrews, and P. Sachdev, *Computer-delivered cognitive behavioural therapy: Effective and getting ready for dissemination.* F1000 Medicine Reports, 2010. 2(49).

## 36. Spurgeon, J.A. and J.H. Wright, *Computer-assisted cognitive-behavioral therapy.* Current Psychiatry Reports, 2010. 12: p. 547-552.

## 37. Richardson, T., P. Stallard, and S. Velleman, *Computerised cognitive behavioural therapy for the prevention and treatment of depression and anxiety in children and adolescents: A systematic review.* Clinical Child and Family Psychology Review, 2010. 13: p. 275-290.

## 38. Andrews, G., *Utility of computerised cognitive-behavioural therapy for depression.* British Journal of Psychiatry, 2010. 196: p. 257-258.

## 39. Arroll, B. and F. Moir, *Time for a rethink of treatment for patients with depression in primary care.* British Journal of General Practice, 2010. 60: p. 641-642.

## 40. Carroll, K.M. and B.J. Rounsaville, *Computer-assisted therapy in psychiatry: Be brave-its a new world.* Current Psychiatry Reports, 2010. 12: p. 426-432.

## 41. Cartreine, J.A., D.K. Ahern, and S.E. Locke, *A roadmap to computer-based psychotherapy in the United States.* Harvard Review of Psychiatry, 2010. 18(2): p. 80-95.

## 42. Newman, M.G., et al., *A review of technology-assisted self-help and minimal contact therapies for anxiety and depression: Is human contact necessary for therapeutic efficacy?* Clinical Psychology Review, 2011. 31: p. 89-103.
