# Supplementary material for: Meta-review of the effectiveness of computerised CBT in treating depression
Source: BMC Psychiatry. 2011 Aug 12;11:131. doi: 10.1186/1471-244X-11-131 (PMC3180363; doi:10.1186/1471-244X-11-131)
Supplement: Additional file 3 — Included reviews. This file lists included review and provides details of their methodology and scope. [file 1471-244X-11-131-S3.DOC]

## Included reviews

| **Authors / year** | **Intervention** | **Target condition** | **Outcome** | **Data bases searched** | **Dates of search** | **Number of studies included** |
| --- | --- | --- | --- | --- | --- | --- |
| **Kaltenthaler et al., 2002 [1]**  **; 2004[2]** | CCBT | Anxiety and depression | Clinical effectiveness and compare the cost-effectiveness of CCBT with two options: • delivering cognitive behaviour therapy (CBT) by conventional methods • treatment as usual (TAU). | 17 electronic bibliographic | 1966 to September 2001 | 16 studies (11 RCTs) and 5 pilot studies or cohort studies.  13 studies for the cost-effectiveness review  4 sponsor submissions were used in the cost-effectiveness analysis |
| **Kaltenthaler et al., 2006 [3]** | CCBT: | Anxiety, depression, phobias, panic and OCD, | Evaluate effectiveness (clinical, interpersonal and social functioning, quality of life, preference, satisfaction and acceptability of treatment); evaluate cost-effectiveness in comparison with current standard treatments; estimate the possible overall cost in England and Wales. | Fifteen electronic bibliographic databases | 1966 to March 2004 | 13 RCTs (2 of which are academic in confidence) 7 non-RCTs  2 RCTs, 2 non-RCTs |
| **Kaltenthaler, et al, 2008 [4]**  **The same study of 2006, focused on depression.** | CCBT | Anxiety and depressive disorders; | Clinical effectiveness | Fifteen electronic bibliographic databases | No date restriction | 4 studies of three computer software packages met the inclusion criteria |
| **Griffiths and Christensen, 2006 [5]** | self-help Internet interventions | mental disorders and related conditions, included: depression, anxiety, stress, insomnia, headache, eating disorder and encopresis | the effectiveness | PubMed, PsycINFO and Cochrane Register Randomised Controlled Trials | the period prior to December 2004 | 15 studies (16 papers)  Only 3 were related to depression, one was on stress and depression. The rest of the studies were related to other psychological problems. |
| **Spek et al, 2007 [6]** | Internet-based cognitive behaviour therapy (CBT) programmes | Depression and anxiety; | (1) Self-rating scales measuring symptoms of depression or anxiety; and (2) clinician rated scales. | PubMed, PsycINFO, and the Social Science Citation Index | 1990 to February 2006 | 12 RCTs |
| **Andersson 2009 [7]** | Internet and computer based treatments | Depresion with or without anxiety | Effectiveness of treatment | PubMed, PsycInfo, Embase, Cochrane Central Register of Controlled Trials | 1966-2009 | 12 RCTs |
| **García-Lizana and Muñoz-Mayorga 2010 [8]** | Information and communication technology (ICT), including cCBT, educational web pages, and videoconferencing. | Depression | Clinical outcomes, use of resources, or satisfaction | Medline, EMBASE, PsycINFO, Centre for Reviews and dissemination, and The Cochrane Library Controlled Trial Registry databases / plus manual search. | 1997-May 2008 | 10 studies, which evaluated various interventions: cCBT (5 studies), educational web pages, videoconferences |
| **Griffiths et al, 2010 [9]** | internet interventions | depression and anxiety disorders | The outcomes, nature and quality of published RCTs and the availability of effective interventions. | the PubMed, PsycINFO, and Cochrane Central Register of Controlled Trials databases | Since the previous study of the authors (published 2007) till (not precisely mentioned) | 26 trials (described in 29 reports)  8 trials for depression; 2 studies targeted both depression and anxiety |
| **Andrews et al 2010 [10]** | Computerised CBT | Anxiety and depression | Effectiveness, and acceptability | PubMed, Cochrane Database of Systematic Reviews and Register of Controlled Trials, Cinahl, PsycINFO, Medline, Social Sciences Citation Index, Embase | Since the previous study of the authors (published 2009) till end of 2009 | 22 studies (9 from the previous review plus 13 new studies)  6 studies on depression, others were on panic disorder, social phobia and general anxiety disorder. |
| **Wade 2010 [11]** | The internet (excluding stand-alone computer programmes) | Depression and anxiety | Screening, supporting, educating, and treating (efficacy) | Medline | 1998 to 2008 | 70 papers cited, referred to 10 RCTs concerning Internet-based CBT  The scope of the paper is devoted to the different applications of the internet in screening, supporting, educating and treating. |
| **Titov 2011 [12]** | Internet-delivered psychotherapy | Depression | efficacy | Pubmed, PsychInfo, Medline | Previous 18 months plus older reviews were no recent work available | 13 RCTs |

1. Kaltenthaler, E., et al., *A systematic review and economic evaluation of computerised cognitive behaviour therapy for depression and anxiety.* Health Technology Assessment, 2002. **6**(22): p. 1-100.

2. Kaltenthaler, E., G. Parry, and C. Beverley, *Computerized cognitive behaviour therapy: A systematic review.* Behavioural and Cognitive Psychotherapy, 2004. **32**(1): p. 31-55.

3. Kaltenthaler, E., et al., *Computerized cognitive behavior theraphy for depression and anxiety update: A systematic review and economic evaluation.* Health Technology Assessment, 2006. **10**(33): p. 1-70.

4. Kaltenthaler, E., et al., *Computerised cognitive-behavioural therapy for depression: Systematic review.* British Journal of Psychiatry, 2008. **193**(3): p. 181-184.

5. Griffiths, K.M. and H. Christensen, *Review of randomised controlled trials of Internet interventions for mental disorders and related conditions.* Clinical Psychologist, 2006. **10**(1): p. 16-29.

6. Spek, V., et al., *Internet-based cognitive behaviour therapy for symptoms of depression and anxiety: A meta-analysis.* Psychological Medicine, 2007. **37**(3): p. 319-328.

7. Andersson, G. and P. Cuijpers, *Internet-based and other computerized psychological treatments for adult depression: a meta-analysis.* Cognitive Behaviour Therapy, 2009. **38**(4): p. 196-205.

8. Garcia-Lizana, F. and I. Munoz-Mayorga, *Telemedicine for depression: a systematic review.* Perspectives in Psychiatric Care, 2010. **46**(2): p. 119-126.

9. Griffiths, K.M., L. Farrer, and H. Christensen, *The efficacy of internet interventions for depression and anxiety disorders: a review of randomised controlled trials.* Medical Journal of Australia, 2010. **192**(11 Suppl): p. S4-11.

10. Andrews, G., et al., *Computer therapy for the anxiety and depressive disorders is effective, acceptable and practical health care: A meta-analysis.* PLoS ONE, 2010. **5**(10).

11. Wade, A.G., *Use of the internet to assist in the treatment of depression and anxiety: A systematic review.* Primary Care Companion to the Journal of Clinical Psychiatry, 2010. **12**(4): p. e1-e11.

12. Titov, N., *Internet-delivered psychotherapy for depression in adults.* Current Opinion in Psychiatry, 2011. **24**: p. 18-23.
